# Characteristics of Atmospheric Reactive Nitrogen Deposition in Nyingchi City

**DOI:** 10.1038/s41598-019-39855-2

**Published:** 2019-03-15

**Authors:** Wei Wang, Wen Xu, Zhang Wen, Dandan Wang, Sen Wang, Zhiwei Zhang, Yuanhong Zhao, Xuejun Liu

**Affiliations:** 1 College of Resources and Environmental Sciences, China Agricultural University, Beijing, 100193 China; 2 Xizang Agriculture and Animal Husbandry University, Nyingchi, Tibet 860000 China; 3 Laboratory for Climate and Ocean-Atmosphere Sciences, Department of Atmospheric and Oceanic Sciences, School of Physics, Peking University, Beijing, 100871 China

## Abstract

Atmospheric reactive nitrogen (N) deposition has been proven to be an important nutrient input from external environments to forest ecosystems. However, the magnitude of atmospheric N deposition in the Tibetan region of China is not well known. In this study, multi-year (between 2005 and 2016) measurements of dry and wet N deposition were carried out in Nyingchi (NC) city, southeastern Tibet. Bulk deposition was collected by the rain gauge method; dry deposition was calculated by the inferential method, namely, multiplying ambient N concentrations by dry deposition velocity (V_d_) of the N species. During the entire period, annual bulk and dry N deposition fluxes averaged 2.19 and 1.85 kg N ha^−1^ yr^−1^, respectively. Total N deposition fluxes (the sum of reduced and oxidized N species in dry and bulk deposition) showed an obvious increasing trend, especially for oxidized N species. Both bulk and dry N deposition showed a consistent seasonal pattern, with the highest fluxes in summer and the lowest in winter. Our findings suggest that N deposition to the urban environment in southeast Tibet has recently shifted from ammonium-dominated to nitrate-dominated conditions.

## Introduction

Atmospheric reactive nitrogen (N) deposition depends mainly on emissions of N species (e.g., NH_3_ and NO_x_), and significantly alters the global N cycle^1^. Although both natural (e.g., soil microbial nitrification and denitrification) and anthropogenic (e.g., nitrogen fertilizer application and fossil fuel combustion) sources contribute to atmospheric N deposition levels, the latter has played a dominant role since the Industrial Revolution. Atmospheric N emission has increased from less than 50 Tg in 1950 to more than 200 Tg in 2000 on a global scale, and from 10 Tg in 1970 to more than 50 Tg in 2010 in China^2–4^. With the increased anthropogenic atmospheric N emissions, the global atmospheric N deposition flux has increased from 31.6 Tg N yr^−1^ in 1860 to 102.5 Tg N yr^−1^ in 1993 and will continue to increase to 194.5 Tg N yr^−1^ in 2050^5^. A number of studies demonstrated that the amount of N deposition increased significantly over recent decades in China; for example, Liu *et al*.^6^ reported a 60% increase in bulk N deposition, from 13.2 kg N ha^−1^ yr^−1^ in the 1980s to 21.1 kg N ha^−1^ yr^−1^ in the 2000s. Similar reports of increased N deposition in China also exist^7,8^.

The Qinghai-Tibet Plateau has N nutrition restricted ecosystems which are sensitive to enhanced nitrogen deposition^9–11^. For example, increased N deposition enhances plant growth and stimulates aboveground N and carbon (C) storage except for legume species^12,13^. Based on a meta-analysis of publications for low N deposition regions, LeBauer and Treseder^14^ found that aboveground net primary productivity increased by 1.2 times in temperate forests and by 1.6 times in tropical forests with N addition compared with corresponding forest types without N addition treatment. So far, only a few studies have focused on the quantification of N deposition fluxes in the Qinghai-Tibet Plateau^8,15,16^. For example, N deposition fluxes were estimated at ~12 kg N ha^−1^ yr^−1^ over the whole region^8,15^. At the single site scale, wet N deposition fluxes were from 0.44 to 1.55 kg N ha^−1^ yr^−1^ at five remote sites in Tibet^17^, whilst in the population concentrated areas, such as Lhasa, N deposition fluxes have reached up to 20 kg N ha^−1^ yr^−1^ ^16^. These results indicate that the spatial distribution of N deposition in Tibetan Plateau varies largely and could be enhanced by anthropogenic disturbance. To date, the magnitude and annual trend of N deposition levels in different ecological areas of Tibet are still unclear, especially in the forest areas of southeastern Tibet.

Nyingchi (NC) city is located in the southeastern Qinghai-Tibet Plateau and Tibet southeast gorge forest area, which accounts for 80% of the total forest area (1.47 × 10^7^ ha) in Tibet Autonomous Region. To estimate dry and bulk N deposition (wet plus part of dry deposition) in southeast Tibet, we established a long-term *in situ* N deposition monitoring site in NC City, a background forested site witnessing the rapid development of the Tibetan economy. The objectives of the present study were to (1) quantify the magnitudes of dry and bulk N deposition fluxes, and (2) investigate the monthly and annual patterns of wet and dry deposition fluxes of various N species.

## Results

### Monthly N concentrations in rainwater and bulk deposition fluxes in NC City

**Figure 1:**
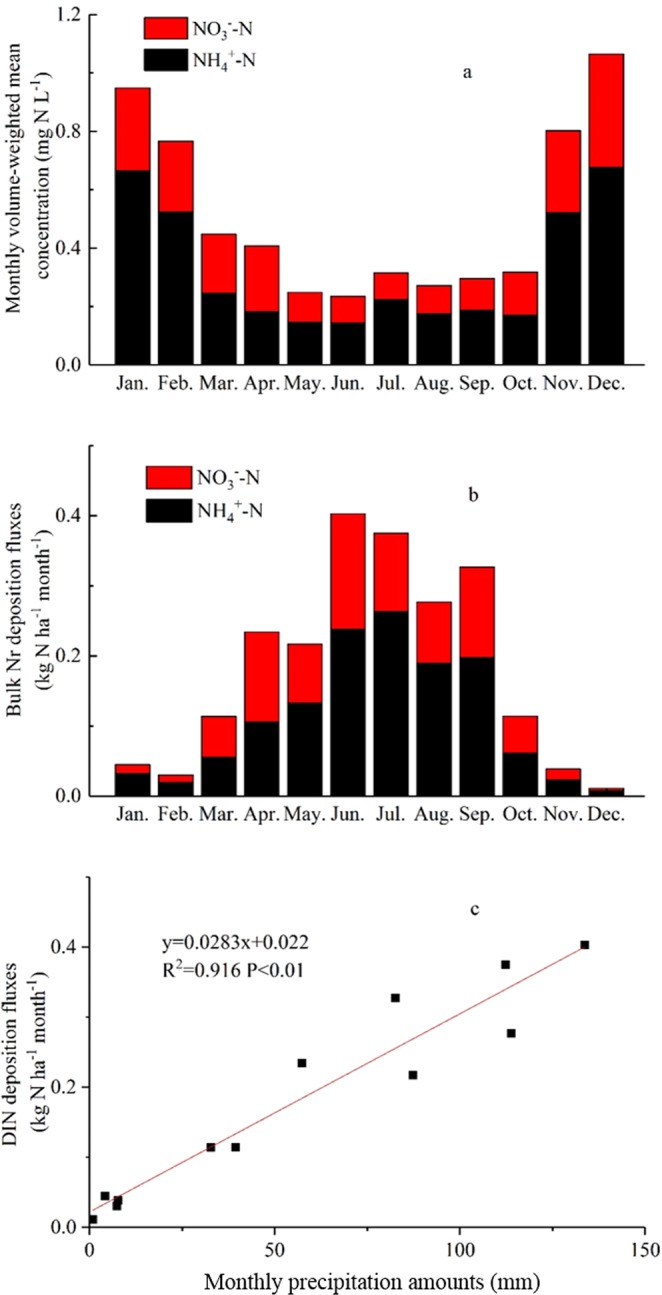
Monthly volume weighted mean concentration (**a**) and bulk deposition fluxes (**b**) in Nyingchi city; the correlation between bulk Nr deposition flux and precipitation amounts (**c**).

Monthly volume-weight mean concentrations were 0.14–0.68 mg N L^−1^ for NH_4_^+^ and 0.09–0.39 mg N L^−1^ for NO_3_^−^ in rainwater (Fig. 1a). Monthly bulk deposition fluxes of NH_4_^+^ and NO_3_^−^ were in the ranges of 0.01–0.26 kg N ha^−1^ and 0.01–0.16 kg N ha^−1^, respectively (Fig. 1b). Bulk N concentrations showed an obvious seasonal variation, with the lowest values in summer and the highest in winter. By contrast, bulk deposition fluxes showed an opposite seasonal behaviour to concentrations.

### Monthly atmospheric N concentrations and dry N deposition fluxes in NC city

**Figure 2:**
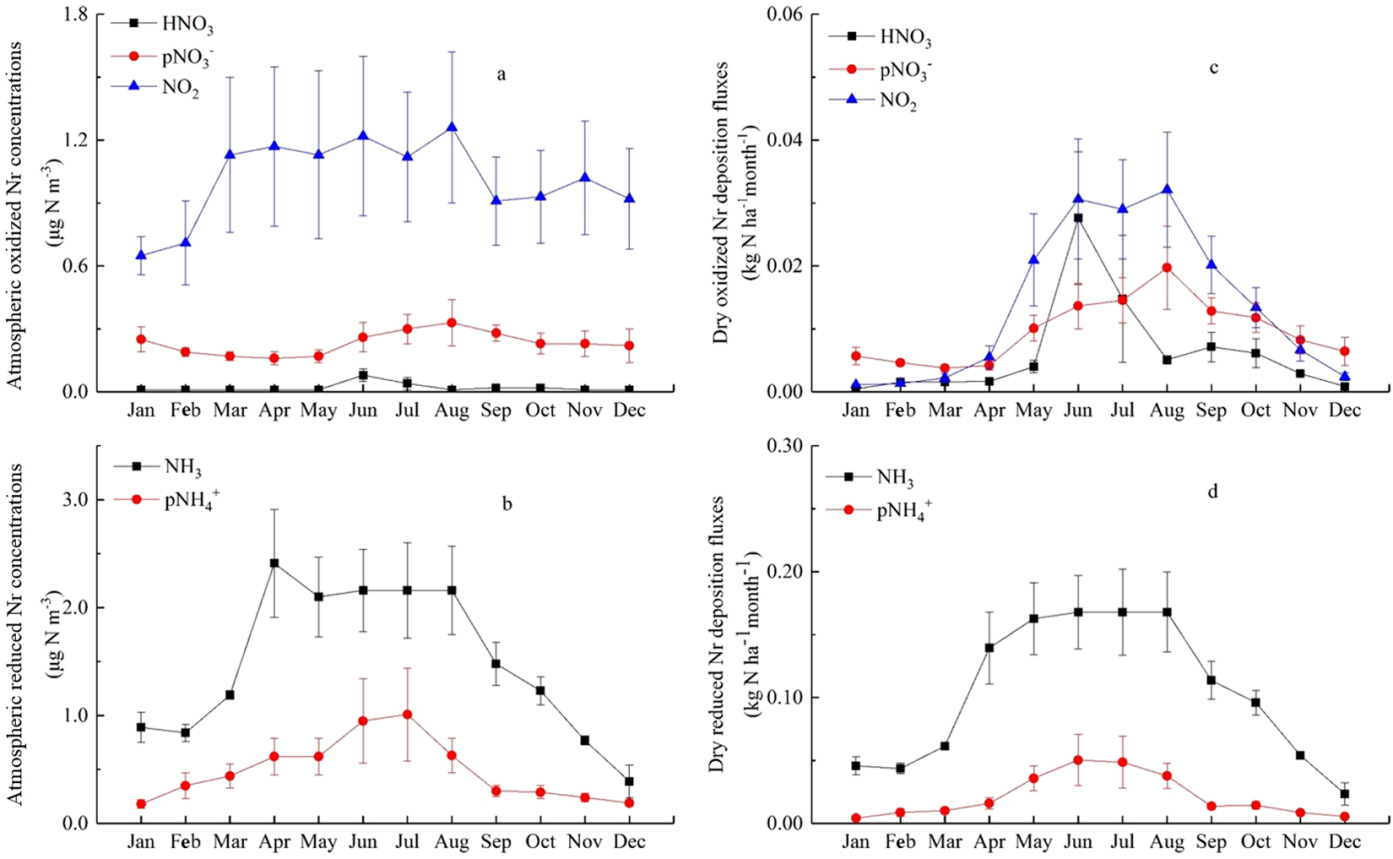
Monthlyoxidized N (**a**) and reduced N concentrations (**b**) in Nyingchi city; dry oxidized N (**c**) and reduced N deposition fluxes (**d**) in Nyingchi city; Error bar in meander line denotes standard error of means.

Monthly mean concentrations of atmospheric NH_3_, HNO_3_, *p*NH_4_^+^, *p*NO_3_^−^, NO_2_ were been monitored over three years (2009, 2015 and 2016) (Fig. 2a,b). Monthly mean concentrations of NH_3_ and *p*NH_4_^+^ were in the ranges 0.39–2.41 and 0.18–1.01 μg N m^−3^, respectively. The highest monthly NH_3_ concentration appeared in April, approximately 6 times higher than the lowest value observed in December. The highest monthly *p*NH_4_^+^ concentration occurred in July, approximately 5 times higher than the minimum value in January. Monthly mean concentrations of NO_2_, *p*NO_3_^−^ and HNO_3_ were in the ranges of 0.65–1.26, 0.16–0.33 and 0.01–0.08 μg N m^−3^, respectively, and the highest monthly concentrations of NO_2_, *p*NO_3_^−^ and HNO_3_ appeared in August, August and June, respectively. The lowest monthly concentrations of NO_2_, *p*NO_3_^−^ and HNO_3_ were observed in January, April, and December, respectively.

Monthly deposition fluxes of NH_3_ and *p*NH_4_^+^were in the ranges 0.02–0.17 and 0.01–0.05 kg N ha^−1^, respectively. Monthly deposition fluxes NO_2_, *p*NO_3_^−^ and HNO_3_ were in the ranges 0.01–0.03, 0.01–0.02 and 0.01–0.02 kg N ha^−1^. Both reduced N and oxidized N deposition fluxes showed an increasing trend in the front six months and a decreasing trend in the later six months in a year. Furthermore, NH_3_ deposition fluxes dominated reduced N deposition in all months, but oxidized N had no unique variations, NO_2_ deposition fluxes were the main oxidized N species from April to October and *p*NO_3_^−^ deposition fluxes was main oxidized N species in January, February, March, November and December (Fig. 2c,d).

### Annual trend of atmosphere N deposition fluxes

**Figure 3:**
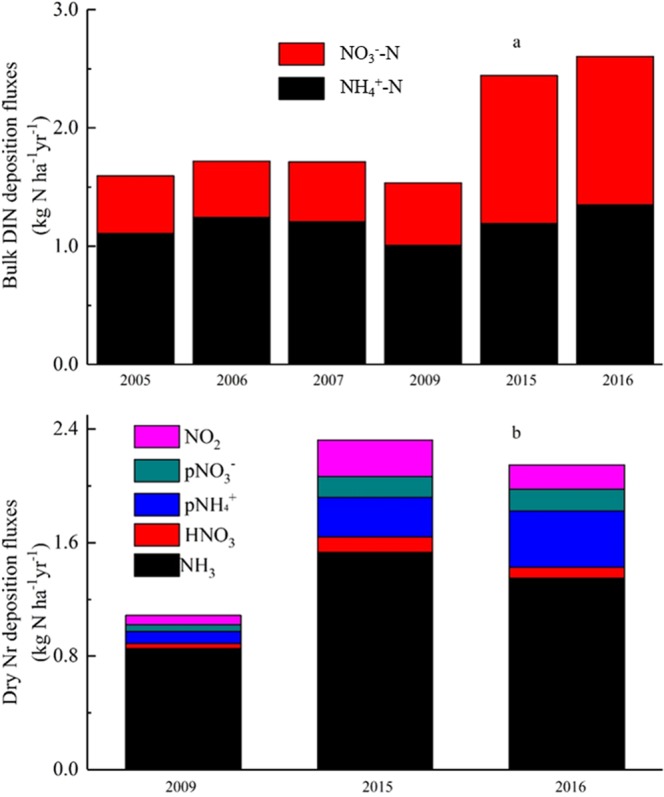
Annual bulk (**a**) and dry (**b**) N deposition fluxes.

Total bulk N depositions fluxes were 1.60, 1.72, 1.71, 1.54, 2.44 and 2.60 kg N ha^−1^ yr^−1^ in 2005, 2006, 2007, 2009, 2015 and 2016, respectively (Fig. 3a). Bulk NH_4_^+^ and NO_3_^−^ deposition fluxes have increased by 1.3 times (from 1.11 to 1.35 kg N ha^−1^ yr^−1^) and 2.6 times (from 0.49 to 1.25 kg N ha^−1^ yr^−1^), respectively, during the entire period.

Total dry N deposition fluxes were 1.09, 2.32 and 2.15 kg N ha^−1^ yr^−1^ in 2009, 2015 and 2016 (Fig. 3b), respectively. Across the 3 years, the deposition fluxes of each N species increased to a varying extent, approximately 1.6 times for NH_3_ (from 0.85 to 1.35 kg N ha^−1^ yr^−1^), 2.1 times for HNO_3_ (from 0.04 to 0.08 kg N ha^−1^ yr^−1^), 4.7 times for *p*NH_4_^+^ (0.08 to 0.39 kg N ha^−1^ yr^−1^), 3.2 times for *p*NO_3_^−^ (0.05 to 0.15 kg N ha^−1^ yr^−1^), 2.5 times for NO_2_ (0.07 to 0.17 kg N ha^−1^ yr^−1^).

### Relative N deposition source attribution

**Figure 4:**
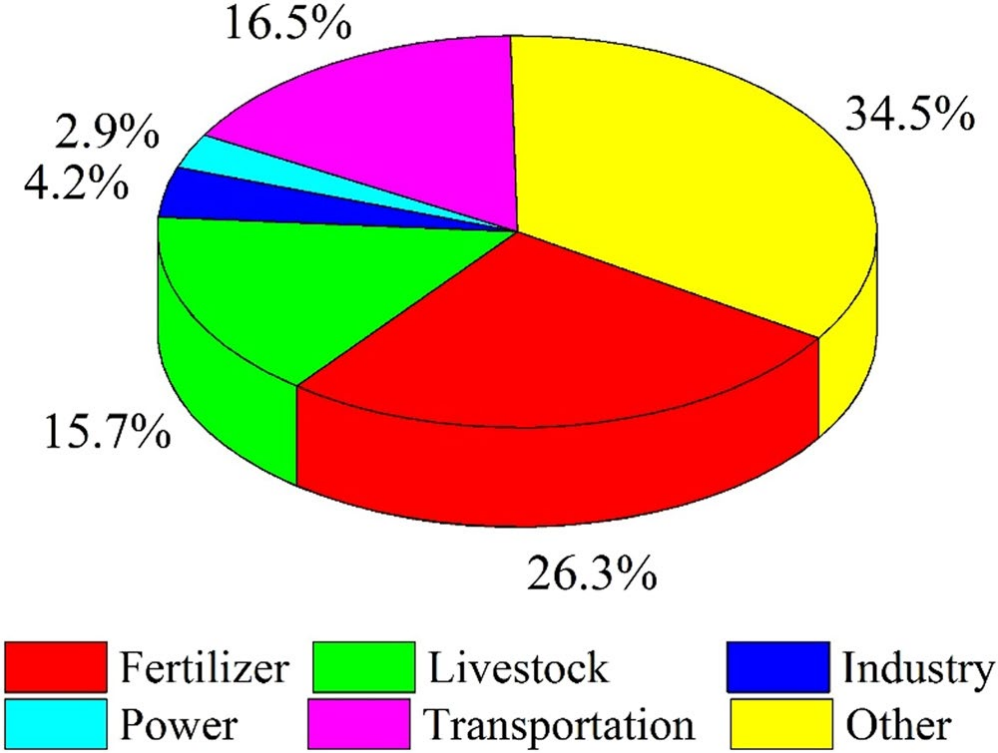
The contributions of N deposition sources to total N deposition in Nyingchi city.

Fertilizer use, transportation and livestock were contributed largely to total N deposition, with the percentages of 26.3%, 16.5% and 15.7%, respectively. Other sources, including human waste, residential activities, soil, lighting and biomass burning, altogether contributed 34.5% of the total N deposition. Industry and power had minor contribution to total N deposition, with percentages of 4.2% and 2.9% (Fig. 4).

## Discussion

Lots of studies have revealed that the lowest concentrations of N species in bulk precipitation are associated with the highest precipitation rates in summer^18,19^, furthermore, atmospheric N bulk deposition fluxes are positively correlated with local precipitation^20^. Monthly precipitation and bulk DIN deposition fluxes were found to be positively correlated (R^2^ = 0.976, P < 0.01) (Fig. 1c). Furthermore, bulk DIN deposition fluxes summed 2.06 kg N ha^−1^ from March to October (Fig. 1b), which accounted for 94% of annual bulk DIN deposition, suggesting that bulk deposition mainly occurred in the plant growth season.

Seasonal variations of atmospheric N species concentrations have been investigated in many studies^18,19,21^. High atmospheric NH_3_ concentrations in summer could be a result of agricultural activities (e.g., N-fertilization) and seasonal changes in temperature^22,23^. Unlike NH_3_, NO_2_ concentrations exhibited a weaker seasonal variation, because they mainly come from industrial and transportation emissions which are non-seasonal emission sources^24^. Atmospheric NH_3_ concentrations were relatively high from April to August (Fig. 2a), which corresponds to local agriculture activities. It is interesting that ambient NO_2_ concentrations were obviously lower in January and February (Fig. 2b), in which relatively higher NO_2_ concentrations were found in other regions of China^20^. This is because, on one hand, there was no winter domestic heating in NC City. On the other hand, the city focused on tourism and there were few people in winter. As a result, transportation emissions as the main NO_2_ source were low in these two months.

Zhu *et al*.^16^ reported that the average wet N deposition flux was 13.69 kg N ha^−1^ yr^−1^ in China. Our measured bulk N deposition flux in Nyingchi city was 2.19 kg N ha^−1^ yr^−1^ (Fig. 3a), just one-sixth of the national level. Not surprisingly, wet N deposition fluxes were influenced by precipitation and different kinds of atmospheric N concentrations^20^. It has been commonly accepted that atmospheric N concentration can be largely affected by anthropogenic activities^9^; the Qinghai-Tibet plateau was considered a region of less human disturbance and a low N deposition area^9^. It could be seen that both NO_3_^−^ and NH_4_^+^ concentrations in rainwater were much lower than in other areas (Table 1), partially resulting in lower N deposition fluxes. At a large spatial scale, Liu *et al*.^17^ reported that wet N deposition fluxes in the Tibetan Plateau were 0.51 kg N ha^−1^ yr^−1^ for NO_3_^−^ and 1.06 for kg N ha^−1^ yr^−1^ NH_4_^+^.

**Table 1:** Comparison of wet/bulk N deposition fluxes measured in different regions.

Site	Concentration (mg*L^−1^)		Deposition fluxes (kg N ha^−1^)		Reference
	NO_3_^−^	NH_4_^+^	NO_3_^−^	NH_4_^+^	
Yangtze			7.1	15.5	^25^
Jiaozhou Bay	3.9	1.93	7.63	13	^26^
Jiangxi	0.25–2.29	0.26–2.06	2.03	6.36	^27^
Chongqing	2.9	1.75	8.16	17.49	^28^
Sichuan	1.05	1.48	6.16	9.92	^18^
Huang-huai -hai plain			3.4	7.78	^29^
Tibet			0.51	1.06	^17^
Feiyue	0.52	0.84	6.9	11.3	^30^
Huinong	0.4	0.68	5.7	9.7	
Xishan	0.57	0.83	7.3	10.7	
Payalaber.Singapo	0.3	0.17			^31^
Cavanagh.Singapo	0.3	0.23			
Kallang.Singapo	0.22	0.17			
Jiangsu	1.3	1.3	14	13	^32^
Liaohe	0.89	1.82	4.7	9.8	^33^
Nyingchi	0.13	0.2	0.86	1.33	This study

The mean concentration and dry deposition fluxes of NH_3_, HNO_3_, *p*NH_4_^+^, *p*NO_3_^−^ and NO_2_ were all lower than the mean values reported across China^20^. Regions with high atmospheric N concentrations were usually coupled with intensive agricultural activities, high population density and a rapidly developing economy. However, the differences of N deposition fluxes in different regions are due to large differences in economic function, weather condition and land use type^34^. Compared with concentrations and deposition fluxes in other areas (Table 2), all types of N species were much lower than in other regions. In addition, NH_3_ and NO_2_ dominated atmospheric N in NC city, the same as in other areas^5^.

**Table 2:** Comparison of concentrations and deposition fluxes of atmospheric N.

Site	Land use	Concentration (μg N m^−3^)					Deposition fluxes (kg N ha ^−1^ yr^−1^)					Reference
		NH_3_	NO_2_	HNO_3_	*p*NH_4_^+^	*p*NO_3_^−^	NH_3_	NO_2_	HNO_3_	*p*NH_4_^+^	*p*NO_3_^−^	
Chengdu	U	12.20	15.40		5.90	5.50						^35^
Wanzhou	U	9.80	12.30	0.25	3.80	0.94						^36^
Payalaber.Singapo	U				0.22	0.62						^31^
Cavanagh.Singapo	U				0.25	0.64						
Kallang.Singapo	U				0.27	0.48						
Shifang	S	14.90	10.60		5.10	3.50						^37^
Europe	SV	1.12	2.39	0.20	0.82	0.40						^35^
Europe	G	2.04	3.54	0.23	0.78	0.52						
Yangtze	F/A						4.50	0.27	2.20	1.90	0.88	^25^
Xishan	F	2.10	6.10	0.60	4.40	1.20	10.50	3.10	6.10	11.20	4.00	^30^
Europe	F	1.03	2.15	0.24	0.73	0.45						^35^
Huinong	A	3.20	4.10	0.70	4.20	0.90	1.40	1.50	1.80	2.00	0.50	^30^
Feiyue	A	5.50	6.00	0.60	4.50	1.10	6.50	1.90	1.70	1.70	0.40	
Europe	A	3.65	2.56	0.30	1.41	0.66						^35^
Yangling	A	6.00	7.20	1.30	3.80	4.10	7.40	3.80	3.20	2.50	2.30	^19^
Sichuan	A	3.71	2.62	0.56	3.15	1.15	2.68	1.08	1.28	1.62	0.59	^18^
Huang-huai -hai plain	A									10.20	3.30	^29^
Yanting	A	4.90	2.60		3.00	2.00						^37^
Jiangsu	A	4.50	42.20				3.20	4.40				^32^
Quzhou	A	14.50	9.30	0.60	13.50	5.30	22.20	17.30	3.70	10.20	4.00	^38^
Dongbeiwang	A	9.50	16.50	0.60	7.10	4.00	10.50	30.70	3.70	5.40	3.00	
Linzhi	U	1.48	1.02	0.02	0.49	0.23	1.24	0.17	0.07	0.25	0.12	this study

Note: U represents urban; S represents suburban; SV represents short vegetation, G represents grass land; F represents Forest; A represents agriculture.

With rapid economic development, NH_3_ and NO_x_ emissions increased continuously and N deposition fluxes were enhanced on a national scale^6,9^. Compared with dry N deposition fluxes in 2009, they increased significantly in 2015 and 2016 (Fig. 3b) in NC city; furthermore, deposition fluxes of oxidized N increased more obviously than that of reduced N from 2009 to 2016. We used statistics from National Bureau of Statistics of Tibet to estimate the possible N source in this study (Table 3). The amount of agricultural fertilizer application and agricultural N fertilizer application, as the main NH_3_ source, increased 1.34 × 10^4^ t and 0.38 × 10^4^ t from 2009 to 2015, an approximately 28% and 22% increase, respectively; urban population increased from 0.66 million to 0.9 million, an approximately 36% increase; vehicles were considered to be the largest source of NO_x_, private cars and operated cars (bus or truck) increased 17.38 × 10^4^ and 2.75 × 10^4^, an approximately 186% and 123% increase. The GAINS model (http://www.iiasa.ac.at/) showed that NH_3_ pollution was significantly higher than NO_x_ pollution in Tibet as well, but the increased magnitude of NO_*x*_ was higher, NH_3_ emissions increased from 151.3 kt yr^−1^ in 2005 to 176.3 kt yr^−1^ in 2015 in Tibet, with an increase of 16.3%, and NO_x_ increased from 19.6 kt yr^−1^ in 2005 to 30.4 kt yr^−1^ in 2015 in Tibet, with an increase of 55.1%.

**Table 3:** Possible N source changes during 2009 and 2015 in Tibet.

Year	2009	2010	2011	2012	2013	2014	2015
Urban population (k)	660	680	690	700	740	820	900
Amount of agricultural fertilizer application (kt)	46.9	47.4	47.9	49.9	57.0	53.4	60.3
Agricultural nitrogen fertilizer application (kt)	16.6	19.2	14.8	16.9	19.7	20.2	20.4
Emissions of nitrogen oxides (kt)			40.6	44.3	44.3	48.3	52.7
Waste water ammonia emissions (kt)			3.3	3.2	3.2	3.4	3.4
Total nitrogen emissions from wastewater (kt)			5.3	5.7	5.8	6.2	7.3
Private car ownership (k)	93.6	110.3	130.9	152.2	195.4	230.1	267.4
Road operated car (bus or truck) ownership (k)	22.4	22.9	28.3	33.6	40.7	46.9	49.9

Note: All data comes from the National Bureau of Statistics.

The comment in parentheses was units, k = thousand; kt = thousand tone.

Xu *et al*.^25^ reported that fertilizer use and transport were the main N sources in China and Yangtze River basin based on GEOS-Chem (http://geos-chem.org) model; similar results were founded in this study, fertilizer use (26.3%) and transport (16.5%) were the main N deposition source at our monitoring site. In addition, livestock was the main N deposition source in this study. This could be explained by the Nr emission source, as shown in Fig. 4, fertilizer, transportation and livestock were all dominated Nr emission sources at our monitoring site (Table 4). Interestingly, others (including human waste, residential activities, soil, lighting and biomass burning) contribution to total N deposition were reached 34.5%, One explanation was that there was no obvious N pollution source was found around NC City; as a result, natural N sources which are unchangeable to some extent in different areas, tend to be more important. The same result was also found in a previous study^25^.

**Table 4:** Annual total NH_3_ and NO_x_ emissions over Qinghai-Tibet plateau and monitoring site (kg N ha^−1^ yr^−1^).

	Source Type	Qinghai-Tibet plateau	Monitoring site
NH_3_	Fertilizer^a^	0.63	1.73
	Livestock	1.00	0.56
	Human waste	0.08	0.03
	Fuel combustion^b^	0.04	0.01
	Natural	0.12	0.13
	Total	1.88	2.45
NO_x_	Industry	0.05	0.00
	Power	0.06	0.10
	Transportation	0.20	0.26
	Residential	0.02	0.00
	Natural^c^	0.37	0.08
	Total	0.69	0.45

^a^Fertilizer NH_3_ emissions include both chemical fertilizer and manure fertilizer.

^b^Fuel combustion of NH_3_ emissions include emission from power plant, industry, transportation and residential.

^c^Natural NO_x_ emissions include emissions from soil, lighting and biomass burning.

Dry N deposition fluxes were estimated by different N species concentrations multiplied by their deposition velocities in our research. As in previous studies^18,19^, different N species velocities have monthly variations, which were high in summer and low in winner. The same variations were found in this study; furthermore, the V_d_ of NH_3_, HNO_3_, *p*NH_4_^+^, *p*NO_3_^−^ and NO_2_ were in the ranges of 0.20–0.30, 0.27–1.50, 0.09–0.23, 0.09–0.23 and 0.01–0.10 cm s^−1^, respectively. In conclusion, monthly dry N deposition flux variations were determined by monthly variations in both N concentrations and deposition velocity.

Since bulk N deposition was measured directly and gaseous dry N deposition was estimated based on modeled V_d_, bulk N deposition flux was more accurate than dry deposition fluxes in this work. However, the rainfall amounts were so low in some precipitation events that that could not finish a complete chemical analysis could not be achieved (<20 ml), due to the high N concentration in low precipitation events, and bulk N deposition flux might be underestimated in this work. Furthermore, rain gauges were continuously opened during the experiment period, and some dry N deposition fluxes were included in bulk N deposition estimations. Clearly, if missing rainfall samples were calculated and additional dry N deposition fluxes were excluded, the assessment of N deposition flux could be more precise.

For dry N deposition flux measurements, both the collection bucket method and the DELTA system were widely used. Dry N deposition fluxes collected by the former were lower than the latter because of the particle NH_4_NO_3_ volatilization^39,40^. However, the particle cut-off size for the DELTA system was 4–5 μm, and large particulate matter of diameter >10 mm is deposited by gravity, defined as sedimentary deposition^41^. In seasons with less rainfall, large atmospheric particles were suffused and dry N deposition collected by a bucket could be more effective. In addition, NH_3_ flux is bi-directional, often both emitted from and deposited to land or plant canopy, and net deposition flux is hard to accurately determine in this experiment. Moreover, modeled dry deposition fluxes are very uncertain and can seldom be compared to measurements^40^. Deposition velocities of different N species were influenced by local meteorological conditions and the underlying surfaces; the meteorological conditions in NC City frequently changed and the microclimate was complex, which bring trapped for model V_d_ estimation in our research area.

Total N deposition fluxes reached 4.75 kg N ha^−1^ yr^−1^ in NC city in 2016, it is no doubt that atmospheric N pollution in NC city was not serious. However, it great attention should be paid to the fact that N had the highest efficiency when atmospheric N deposition was between 4 and 10 kg N ha^−1^ yr^−1^ for plant growth and carbon sequestration^15^. Numerous simulated N deposition experiments have been conducted in forest ecosystems and have shown that additional N input could promote plant growth and carbon sequestration^14,42–44^; when total N deposition ranged between 3–11 kg N ha^−1^ yr^−1^, the annual above-ground growth of surviving trees and net annual above-ground carbon increment increased approximately 0.6 t C ha^−1^ yr^−1^, a nearly 5.5% increase per kg N ha^−1^ yr^−1^ ^45^. Magnani *et al*.^46^ also suggested that net ecosystem production in forests showed small changes when N deposition flux was lower than 3 kg N ha^−1^ yr^−1^; however, that value could be increased from less than 1 t C ha^−1^ yr^−1^ to more than 4 t C ha^−1^ yr^−1^ when N deposition fluxes ranged from 3–9 kg N ha^−1^ yr^−1^. As total N deposition flux reached 4.75 kg N ha^−1^ in 2016 and might continue increasing in coming years, our results suggested that more carbon could be fixed in southeast Tibet.

In conclusion, a long-term N deposition monitoring site was established in NC City to measure dry deposition for 3 years (2009, 2015–16), and bulk deposition for 6 years (2005–07, 2009, 2015–16). Averaged bulk N deposition flux was 2.19 kg N ha^−1^ yr^−1^ and averaged dry deposition flux was 1.85 kg N ha^−1^ yr^−1^ in NC City. Atmospheric N concentration and deposition flux were largely enhanced. Both atmospheric reduced N and oxidized N concentration increased with local economic development. Although N deposition was still dominated by reduced N, oxidized N deposition fluxes increased more obviously. In general, atmospheric N deposition flux was 4.75 kg N ha^−1^ yr^−1^ in NC City in 2016, and the influence of N deposition on the local forest ecosystem should be given attention, as much as plant growth and carbon sequestration.

## Materials and Methods

### Site description

**Figure 5:**
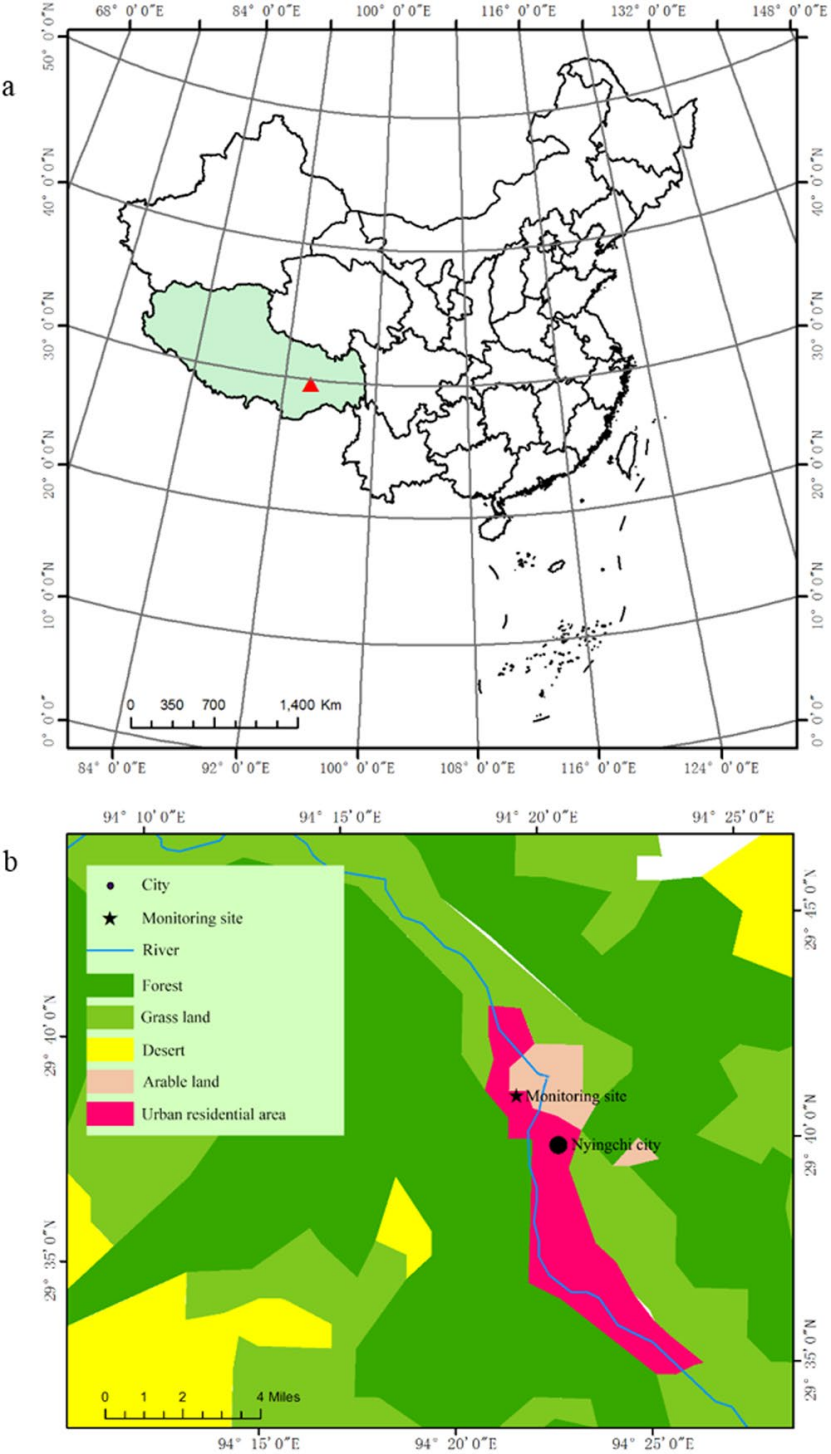
Location (**a**) and land use type (**b**) of experiment monitor site at Nyingchi city, Qinghai-Tibet plateau.

The monitoring site was located at Xizang Agriculture and Animal Husbandry College (29.66°N 94.34°E 2990 m a.s.l.), NC city (Fig. 5). NC City is located beside the Niyang River, which is one of the main tributaries of the Brahmaputra. The climate is mainly dominated by the Indian Ocean and the Pacific warm current. The regional climate is the cold temperate humid subalpine zone, with weather data presented in Table 5. Tourism is the local economic pillar industry. There was no heavy industry nearby, and potential emission sources were small villages and agricultural fields.

**Table 5:** Meteorological data in Nyingchi city.

Month	Jan	Feb	Mar	Apr	May	Jun	Jul	Aug	Sep	Oct	Nov	Dec
Precipitation (mm)	1.3	4.4	19	46.2	75.3	119.2	143.3	122.2	110.5	45.4	4.7	1
Atmospheric pressure (hPa)	708.2	707.7	708	709	708.8	707.9	707.9	709.4	711.1	712.3	711.6	710.4
Temperature (°C)	1	2.8	5.9	8.9	12.1	15.1	16.2	15.7	13.9	10.3	5.5	1.7
Humidity (%)	49	53	58	63	65	72	76	76	76	66	56	50

Note: All data are average values between 1981 and 2010 from the China Meteorological Data Network, available online: http://data.cma.cn/data/weatherBk.html.

### Collection of N compounds in air

Ambient concentrations of gaseous (NH_3_, HNO_3_, NO_2_) and particulate (*p*NH_4_^+^ and *p*NO_3_^−^) N compounds were measured for three years (2009, 2015 and 2016) using an active sampling system (DEnuder for Long Term Atmospheric sampling-DELTA)^41^. The system was based on a set of bore glass denuder traps through which a laminar flow of air is driven by a pump (pumping rates were 0.3–0.4 L min^−1^) set at the end of the system. A first set of denuders was coated with citric acid to capture gaseous NH_3_, and a second set was coated with potassium hydroxide to trap gaseous HNO_3_. Aerosols pass through the denuders without reacting and then are collected by a couple of filters placed downstream of the denuders. These filters were treated with the same alkaline (for *p*NH_4_^+^ sampling) and acid (for *p*NO_3_^−^ sampling) solutions as the denuders. Ambient NO_2_ concentrations were measured using Gradko passive diffusion tub with three replicates. The DELTA system and Gradko NO_2_ tubes were placed at a height of 1.5 m above the ground, and these samplers were exposed to ambient air for one month (one sample per month). After sampling, exposed DELTA sampling train and NO_2_ diffusion tubes were returned to the laboratory for sample analysis.

### Precipitation collection

A rain gauge was installed beside the DELTA to measure monthly bulk deposition, at a height of 1.5 m above ground level. Rainfall was collected immediately after each precipitation event, and then transferred into 100 ml polyethylene bottles. The precipitation samples were thoroughly mixed and transferred to clean polyethylene bottles (50 ml), and then stored at −20 °C until chemical analysis within one month. After collection, the rain gauge was rinsed with deionized water. The measurements were performed for the 6 years (2005–2007, 2009, 2015, and 2016).

### Analytical procedures

The exposed DELTA sampling trains and NO_2_ diffusion tubes were sent to China Agriculture University after each monthly collection was finished, then stored at 4 °C and analysed within one month. Different N species were extracted by different solutions, gaseous NH_3_ was captured and *p*NH_4_^+^ in alkaline filters in the DELTA system were extracted by 0.05% H_2_O_2_ solution; gaseous HNO_3_ was captured and *p*NO_3_^−^ in acid filters in the DELTA system were extracted by high-purity water. Nitrate (NO_3_^−^) and ammonium (NH_4_^+^) in the extracted and filtered solutions were measured by an AA3 continuous-flow analyser (Bran + Luebbe GmbH, Norderstedt, Germany), the detection limits of NH_4_^+^ and NO_2_^−^ were 0.01 mg N L^−1^. The blank samples were also analyzed to eliminate the errors in the experimental process.

The stainless steel mesh in the Gradko passive diffusion tube was leached with a mixed solution of sulfonamide, phosphoric acid and N-1-naphthylethylen-diamina (NEDA), and the concentration of NO_2_^−^ was determined by colorimetry at a wavelength of 542 nm. The blank sample was analysed in the same way to eliminate errors during the experiment.

Precipitation samples were filtered with a 0.45 µm syringe filter before determination each month. NH_4_^+^ and NO_3_^−^ concentrations in the filtrate were measured using the AA3 continuous-flow analyser as described above.

### Estimation of bulk N deposition

The volume-weighted mean of the monthly concentrations of NO_3_-N and NH_4_^+^-N in bulk precipitation were calculated separately using the following equation:

$${C}_{w}=\sum _{i=1}^{n}{C}_{i}\times {P}_{i}/\sum _{i=1}^{n}{P}_{i}$$

where *C*_*w*_ refers to the volume-weighted mean concentrations of NO_3_^−^-N and NH_4_^+^-N in bulk precipitation (mg N L^−1^); *C*_*i*_ is the NO_3_^−^-N and NH_4_^+^-N concentration in bulk precipitation for each individual sample (mg N L^−1^); *P*_*i*_ is the amount of precipitation corresponding to each sample (mm); and *n* refers to the number of samples. Mean monthly of NO_3_^−^-N and NH_4_^+^-N deposition fluxes were calculated as follows:

$${D}_{w}={P}_{t}\times {C}_{w}/100$$

where *D*_*w*_ refers to the mean monthly bulk NO_3_^−^-N or NH_4_^+^-N deposition (kg N ha^−1^ month^−1^); *C*_*w*_ is the monthly volume-weighted mean concentration of NO_3_^−^-N or NH_4_^+^-N in bulk precipitation (mg N L^−1^); *P*_*t*_ is the mean monthly precipitation (mm); 100 is the unit conversion factor.

### Estimation of dry N deposition flux

Dry deposition fluxes were estimated by different kinds of atmospheric N concentrations multiplied by their deposition velocities (V_d_), as has been widely done in previous studies^20,21^. Monthly averaged atmospheric N concentrations species measurements were as described above. Monthly averaged V_d_ was calculated by the GEOS-Chem chemical transport model (http://geos-chem.org)^47^. The GEOS-Chem CTM is driven by GEOS-5 assimilated meteorological data from the NASA Global Modeling and Assimilation Office (GMAO) with a horizontal resolution of 1/2° latitude × 2/3° longitude and 6 h temporal resolution (3 h for surface variables and mixing depths)^20^.

### Source attribution of N deposition

The GEOS-Chem (http://geos-chem.org) model was used to estimate the emission sources of N deposition in NC City, which has been widely used to assess the contribution of different N emission source across China^26,48^. The Nest GEOS-Chem atmospheric chemistry model was used to test the model sensitivity driven by the GEOS-5 assimilated meteorological fields at a horizontal resolution of 1/2° × 2/3°. Based on the Multi-Resolution Emission Inventory of China in 2010 (MEIC, http://meicmodel.org), anthropogenic emissions except NH_3_ were used in this research; NH_3_ emissions were based on the Regional Emission in Asia (REAS-v2) inventory and improved by Zhao *et al*.^48^. Details of the model emissions and mechanisms have been provided in Zhao *et al*.^48^. The NH_3_ and NO_x_ emissions over the Qinghai-Tibet pleateau are 1.88 kg N ha^−1^ yr^−1^ and 0.69 kg N ha^−1^ yr^−1^ in 2010. Fertilizer and livestock were dominant NH_3_ sources, accounting for 34% and 53% of the NH_3_ emissions, respectively. Natural and transportation were main NO_x_ sources, accounting for 54% and 29% of total NO_*x*_ emissions, respectively. The NH_3_ and NO_x_ emissions were 2.45 and 0.45 kg N ha^−1^ yr^−1^ at our monitoring site, respectively. Fertilizer (71%) was the donimated NH_3_ emission source and transportation (58%) was the dominant NO_*x*_ emission source (Table 4).

The data on possible N emission sources (urban population; agricultural fertilizer application; emissions of nitrogen oxides; waste water ammonia emissions and total nitrogen emissions from wastewater) were obtained from the National Bureau of Statistics of Tibet (http://data.stats.gov.cn/easyquery.htm?cn=C01).

### Statistical analysis

All statistical analyses were performed using SPSS 20.0 (SPSS Inc., Chicago, IL, USA), with significance level set at P < 0.05. All the data were shown as the mean ± standard error (SE) of corresponding months during the observation period. The periods of March-May, June-August, September-November, and January-December were defined as spring, summer, autumn and winter.
